# Abnormal neural circuits and altered brain network topological properties in patients with chronic unilateral vestibulopathy

**DOI:** 10.1007/s10072-025-08183-x

**Published:** 2025-04-21

**Authors:** Yuru Wang, Kangzhi Li, Lihong Si, Shui Liu, Xu Yang

**Affiliations:** 1 Department of Neurology, Peking University Aerospace School of Clinical Medicine, Beijing, 100049 People’s Republic of China; 2 Department of Neurology, Peking University Shougang Hospital, Beijing, 100144 People’s Republic of China; 3 Department of Neurology, Shanxi Bethune Hospital, Shanxi Academy of Medical Sciences, Third Hospital of Shanxi Medical University, Tongji Shanxi Hospital, Taiyuan, 030032 People’s Republic of China; 4 Department of Radiology, Aerospace Center Hospital, Peking University Aerospace School of Clinical Medicine, Beijing, 100049 People’s Republic of China; 5 Department of Neurology, Peking University First Hospital, Beijing, 100034 People’s Republic of China

**Keywords:** Chronic unilateral vestibulopathy, Multisensory integration, Graph-theoretical analysis, Dynamic compensation

## Abstract

**Background:**

Chronic unilateral vestibulopathy (CUVP) is one of the most common causes of chronic dizziness/vertigo. The brain functional mechanisms of CUVP are currently unclear. The study aimed to clarify changes in brain topological properties and subnetwork functional connectivity in CUVP patients, elucidating the neural mechanisms behind their poor dynamic compensation.

**Methods:**

A total of 44 participants were included (22 CUVP patients and 22 age- and sex-matched healthy controls). Resting-state functional MRI was performed on all subjects. Network-Based Statistics (NBS) analysis was conducted to identify abnormal neural circuits in CUVP. Graph-theoretical analysis (GTA) was performed to elucidate changes in brain network topological properties. Correlation analysis was conducted to examine the relationship between brain network changes and clinical symptom severity.

**Results:**

NBS analysis revealed an abnormal neural network in CUVP patients, with key nodes including the parieto-insular vestibular cortex, sensory-motor cortex, occipital visual cortex, brainstem, and cerebellum. The most significant functional connectivity abnormalities were observed between the brainstem and visual/sensorimotor networks. Graph-theoretical analysis indicated increased characteristic path length, decreased global and local efficiency in CUVP patients. Node properties showed reduced node efficiency and clustering coefficients in multiple nodes within the visual and sensorimotor networks. Correlation analysis indicated that brain network topology and changes in brainstem-sensorimotor network connectivity were negatively correlated with DHI scores.

**Conclusion:**

CUVP patients exhibit multisensory integration abnormalities and changes in brain network topology at both the brainstem/cerebellar and cortical levels, which may underlie the potential neural basis for poor vestibular compensation in CUVP patients.

**Supplementary Information:**

The online version contains supplementary material available at 10.1007/s10072-025-08183-x.

## Introduction

The vestibular system is a essential component of the somatosensory system, playing a vital role in maintaining clear vision and postural stability [1]. Damage to vestibular function can impair these abilities, leading to a range of clinical symptoms [2]. Peripheral vestibular disorders provide a valuable model for studying the pathophysiological and compensatory mechanisms associated with vestibular injury. Unilateral peripheral vestibular dysfunction is one of the most common clinical causes of chronic dizziness and vertigo [3].

Based on the onset pattern and disease course, unilateral vestibular hypofunction is typically classified into acute unilateral vestibulopathy (AUVP) and chronic unilateral vestibulopathy (CUVP). Currently, clinical practitioners and researchers focus more on AUVP [4], while CUVP receives insufficient attention. However, a significant portion of patients with AUVP may experience incomplete vestibular compensation, leading to chronic conditions accompanied by dynamic symptoms [5]. CUVP is prone to persistence or recurrence, placing a considerable burden on patients’ daily lives. Furthermore, CUVP serves as a valuable model for studying vestibular compensation in vestibular disorders.

Previous studies have focused on the structural changes in the brains of patients with CUVP. In a study on 22 patients recovering from vestibular neuritis, Peter et al. [6] used voxel-based morphometry (VBM) analysis found increased volume in the bilateral vestibular nuclei and the interconnecting fibers between them. Helmchen et al. [7] reported increased gray matter volume in the bilateral somatosensory cortex in 15 patients with unilateral vestibular lesions following acoustic neuroma resection. Hong et al. [8] found increased gray matter volume in the occipital lobe and lingual gyrus in 9 patients recovering from vestibular neuritis. These studies highlight structural changes in brainstem structures related to vestibular compensation and sensory substitution mechanisms. In our preliminary study [9], we performed resting-state fMRI on right-sided CUVP patients, and found significant abnormal changes in brain spontaneous activity. This may be related to poor dynamic compensation in CUVP patients.

Currently, research on CUVP primarily focuses on structural changes in the brain and local functional activity, with a lack of whole-brain functional connectivity analysis. Whole-brain functional connectivity analysis is particularly important for understanding the abnormal multisensory integration in CUVP, especially the interactions between vestibular, visual, and proprioceptive systems, as well as changes in sensory-motor integration. Additionally, previous analyses of functional connectivity in vestibular diseases have often been limited to the cortical level [10, 11], lacking adequate exploration of changes in brainstem nuclei. Investigating changes in the brainstem/cerebellar-cortical loops in CUVP patients is essential for understanding the central pathophysiological mechanisms of vestibular diseases, which is currently lacking in research.

Furthermore, the human brain is a highly complex and intricately interconnected network capable of separating and integrating information. Vestibular information is a critical component of somatosensory data, and it remains unclear whether the reduction in vestibular function in CUVP patients leads to changes in the efficiency and characteristics of information transmission in the brain. In recent years, graph theory-based complex brain network analysis has garnered increasing attention, revealing the topological properties of brain networks from the perspective of information transmission [12]. This approach allows for elucidating the pathogenesis of brain diseases based on changes in the topological properties of patients’ brain networks and has been widely applied in the study of neuropsychiatric disorders [13].

Against this backdrop, this study aims to characterize the changes in brain topological properties and subnetwork functional connectivity in CUVP patients during resting states through a combination of functional connectivity and graph theory analysis. We hypothesize that UPVD patients exhibit abnormalities in information transmission and graph theory properties in the brain, which may be the neural basis for poor vestibular compensation in such patients. We hope that the changes in topological properties and subnetwork connectivity will provide new insights into understanding the neural mechanisms of CUVP.

## Methods

### Participants

This is a single-center retrospective study. A total of 22 patients with CUVP who sought treatment for vertigo/dizziness at Aerospace Center Hospital between September 2018 and December 2023 were included. CUVP was defined as the presence of chronic, episodic dizziness/vertigo and/or postural instability symptoms lasting for more than three months; vestibular function tests indicating unilateral peripheral vestibular dysfunction; no symptoms or signs of central nervous system involvement; and the exclusion of other causes of chronic dizziness. Following medical history collection, all CUVP patients underwent videonystagmography to exclude spontaneous nystagmus and confirm static vestibular compensation.Patients who underwent vestibular neurectomy were excluded. Vestibular function tests, including caloric test and video head-impulse testing (vHIT) were performed to determine the extent of peripheral vestibular dysfunction. According to caloric test performed with water irrigation, using a hot temperature of 44 °C and a cold temperature of 30 °C, a vestibular response reduction of at least 25% on one side was defined as unilateral peripheral vestibular dysfunction (i.e., CP > 25%). For vHIT tests, a gain lower than 0.79 is considered indicative of semicircular canal dysfunction. Routine head MRI was performed to exclude severe neurological diseases and intracranial lesions, along with laboratory tests like blood counts and biochemistry to rule out other internal diseases. Simultaneously, 22 age- and gender-matched healthy subjects who underwent phyical physical examination in Aerospace Center Hospital during the same period were included; all healthy subjects had no history of headaches or dizziness and no serious internal diseases. All included subjects underwent resting-state fMRI scans. To avoid drug effects on brain activity, all subjects had not taken psychotropic medications and vestibular suppressants for at least one week prior to scanning. All subjects voluntarily participated in this study and signed informed consent forms. This study was approved by the Ethics Committee of Peking University Aerospace School of Clinical Medicine.

### fMRI image acquisition

Participants underwent scanning on a Siemens 3.0-Tesla MRI machine equipped with a 32-channel head and neck coil. Resting-state fMRI data were collected using an EPI sequence (TR = 2000 ms, TE = 30 ms, flip angle = 90°, FOV = 222 × 222 × 222 mm, voxel size = 3.0 × 3.0 × 3.0 mm), capturing 200 volumes. Structural images were acquired through a 3D T1-weighted sequence (TR = 1900 ms, TE = 2.43 ms, flip angle = 8°, voxel size = 1.0 × 1.0 × 1.0 mm, 192 slices). To minimize head movement, a fixation system was employed, and participants wore headphones to reduce noise. They were instructed to remain relaxed, keep their eyes closed, and stay awake throughout the scan.

### Data pre-processing

Data preprocessing was performed using DPABI [13] (version 8.1) in MATLAB 2022. The preprocessing pipeline consisted of several key steps. First, the initial 10 time points were discarded. Next, slice-timing correction and head-motion correction were applied. Participants who exceeded 2 mm of head translation or 2°of rotation were excluded, while mean framewise displacement (FD) was controlled in subsequent analyses. Spatial normalization was then performed using DARTEL. Nuisance regression followed, incorporating 24 motion parameters as well as white matter and CSF signals, but without global signal regression. To exclude the potential confounding effect of global mean signal differences, this study provides validation of the changes in brain network topological properties after regressing out the global mean signal (supplementary Fig. 1). Finally, a bandpass filter was applied, retaining frequencies between 0.01 and 0.1 Hz.

### Functional connectivity matrix construction and NBS analysis

To investigate changes in interactions between the brainstem, cerebellum, and cerebral cortex, templates were used from the Eagle Atlas Compilation [14] (brainstem and thalamus), Nettekoven_2023 [15] (cerebellum), and dosenbach160 atlas [16] (cortex). After excluding redundant nodes in the thalamus and cerebellum, a total of 253 nodes were included in the analysis. BOLD signal time series were extracted for each node, and a 253 × 253 functional connectivity matrix was generated for each subject. NBS analysis was conducted to identify potential abnormal neural circuits in CUVP patients. The analysis used the whole-brain functional connectivity matrices, with age, gender, mean FD as covariates. Our study adhered to the guidelines of current NBS research, setting the primary threshold at *p* = 0.001 to rigorously control for Type I error [17]. The data from each group underwent 5000 randomizations to generate the reference cluster distribution [18]. For each randomization, we used the maximum number of connections across all clusters to construct the reference distribution. Clusters with observed scores exceeding the 95th percentile were considered significant (*p* < 0.05). Network connectivity maps were created to illustrate the altered interactions between brain networks, with nodes categorized by their respective networks.

### Graph theoretical analysis

Graph theoretical analysis was performed using DPABINet [19] (v1.3) to assess both global and nodal properties of the brain network. Given the lack of systematic studies on the topological characteristics of CUVP patients, the analysis focused on functional integration, segregation, and the importance of specific brain regions. Global metrics included global efficiency (Eglob), local efficiency (Eloc), clustering coefficient (Cp), characteristic path length (Lp), and small-worldness (σ), which balances global and local efficiency. Assortativity was evaluated to examine the tendency of similarly connected nodes to link, while modularity assessed the network’s division into distinct subgroups.

For nodal metrics, degree centrality (DC) quantified direct connections, nodal efficiency (Ne) measured local communication efficiency, and nodal clustering coefficient (NCp) evaluated local interconnectedness. Betweenness centrality (BC) assessed a node’s role in information flow, while eigenvector centrality gauged node importance based on the centrality of its neighbors. PageRank and subgraph centrality were used to measure node significance and involvement in subgraph structures. A weighted network approach was employed, and thresholds ranging from 0.10 to 0.34 were tested at 0.01 intervals to account for the lack of consensus on optimal sparsity [20]. The area under the curve (AUC) was calculated for each metric to capture the brain’s functional connectome comprehensively.

### Statistical analysis

Statistical comparisons of network properties were performed in DPABINet [19], with age, gender, mean FD as covariates. Non-parametric permutation tests (5000 permutations) were conducted to assess group differences, with P-values calculated based on the proportion of permutations where observed differences exceeded random group assignments. A significance threshold of *P* < 0.05 was applied, and false discovery rate (FDR) correction was used for multiple comparisons in nodal metrics.

In this study, the CUVP patients included those with left-sided CUVP and right-sided CUVP. The side of the lesion may potentially affect the results. To further control for the impact of lesion laterality on the outcomes, lesion laterality was incorporated as a covariate in regression analysis. Differences between healthy subjects and the CUVP patient group were then compared to ensure reliability.

Pearson correlation analyses were performed to examine the relationships between the abnormal neural circuits identified through NBS, brain network topological property changes, and DHI scores in CUVP patients. FDR correction was applied to account for multiple comparisons in these analyses.

## Results

### Baseline characteristics of CUVP patients and healthy controls

This study included 44 subjects, comprising 22 CUVP patients and 22 age- and gender-matched healthy subjects. Among the 22 CUVP patients, 9 were males and 13 were females, with an average age of 47.41 ± 11.58 years. All patients were right-handed. The median duration of illness was 9 months, with an interquartile range of 3.75 to 27.00 months. Regarding the affected side, 13 patients had reduced vestibular function on the right side, while 9 had reduced function on the left. In terms of clinical manifestations, 7 patients presented with spontaneous vertigo/dizziness after an acute course, 6 patients experienced head movement or body movement-induced vertigo/dizziness, 5 patients had chronic dizziness after an acute course, and 4 patients experienced chronic dizziness following paroxysmal vertigo. All patients had postural instability, and 13 out of the 22 patients had vestibular-visual symptoms.

For vestibular function assessment, all patients underwent the caloric test, with an average CP value of 52.23 ± 23.67%. Of the 22 patients, 19 completed vHIT test. Among these pateints, 10 showed no significant abnormalities, 6 had decreased gain in both horizontal and vertical semicircular canals, 2 had decreased gain in the vertical semicircular canal, and 1 had decreased gain in the horizontal semicircular canal. Eighteen patients initially presenting with an acute course met the diagnostic criteria for AUVP according to the Bárány Society. The causes of vestibular dysfunction included vestibular neuritis (*n* = 9), possible labyrinthine stroke (*n* = 5), membranous labyrinth hydrops (*n* = 3), labyrinthine hemorrhage (*n* = 1 ), and unknown cause (*n* = 4).

The 22 healthy subjects were all right-handed, had an average age of 47.68 ± 11.90 years. There was no significant difference in the average age between the two groups. The average DHI score for UPVD patients and healthy subjects were 36.41 ± 9.94 and 0, respectively.

### Network-based statistic analysis results

**Fig. 1:**
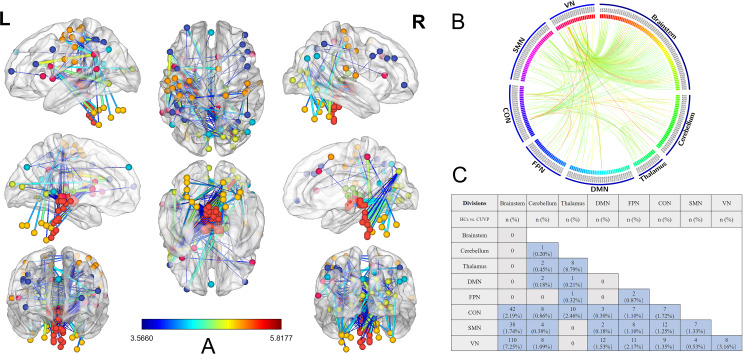
The abnormal neural network identified by network-based statistics. **A**: The abnormal neural network in CUVP patients. **B**: Alterations in functional connectivity among brain networks with the nodes categorized by their networks in CUVP patients; **C**: Quantitative assessment of edges in the abnormal neural circuits revealed by network-based statistic analysis. CUVP, chronic unilateral vestibulopathy; HC, healthy controls; DMN: default mode network; FPN: fronto-parietal network; CON: cingulo-opercular network; SMN: sensorimotor network; VN: visual network

NBS analysis revealed an abnormal neural network in CUVP patients (see Fig. 1A), with the parieto-insular vestibular cortex (PIVC), sensory-motor cortex, occipital visual cortex, brainstem, and cerebellum serving as key nodes in this network. This network consisted of 151 nodes, of which 55 were located in the brainstem, 14 in the cerebellum, 10 in the thalamus, and 72 in the cerebral cortex (including 9 nodes in the default network, 14 in the fronto-parietal network, 14 in the cingulo-opercular network, 18 in the sensorimotor network, and 17 in the visual network).

In total, this network included 327 edges connecting the nodes, categorized based on their respective networks (see Fig. 1B and C). The most connections were found between the brainstem and the visual network (VN), with 110 edges (accounting for 7.25% of its inherent connections). The second most were between the brainstem and the cingulo-opercular network (CON), with 42 edges (accounting for 2.19% of its inherent connections). Among cortical node connections, the most significant functional connectivity changes were observed between the cingulo-opercular network (CON) and the sensorimotor network (SMN), with 12 edges (accounting for 1.25% of its inherent connections). Significant alterations were identified in the PIVC region, precentral gyrus, and premotor area.

There were three patterns of altered functional connectivity in brainstem nuclei: brainstem-VN, brainstem-SMN, and brainstem-CON, with the brainstem-VN showing the most significant changes. Bilateral vestibular nuclei were connected to the left insula (MNI: X=-46, Y = 10, Z = 14), left postcentral gyrus (MNI: X=-24, Y=-30, Z = 64), right lingual gyrus (MNI: X = 19, Y=-66, Z=-1), and right precuneus (MNI: X = 15, Y=-77, Z = 32).

Among cortical nodes, six nodes showed significant connections with the brainstem. These included the left insula (MNI: X=-46, Y = 10, Z = 14), which was connected to 36 brainstem nuclei; the left postcentral gyrus (MNI: X=-24, Y=-30, Z = 64), connected to 33 nuclei; left supramarginal gyrus (MNI: X=-55, Y=-44, Z = 30), connected to 14 nuclei; the left occipital BA30 region (MNI: X=-18, Y=-50, Z = 1), connected to 30 nuclei; the right lingual gyrus (MNI: X = 19, Y=-66, Z=-1), connected to 56 nuclei; and the right precuneus (MNI: X = 15, Y=-77, Z = 32), connected to 27 nuclei.

The network also included 14 cerebellar nodes with 25 edges, of which 8 connected to the visual network, 8 to the cingulo-opercular network, 4 to the sensorimotor network, 2 to the thalamus, 2 to the default network, and 1 was internal cerebellar connections.

### Graph theoretical analysis results

**Fig. 2:**
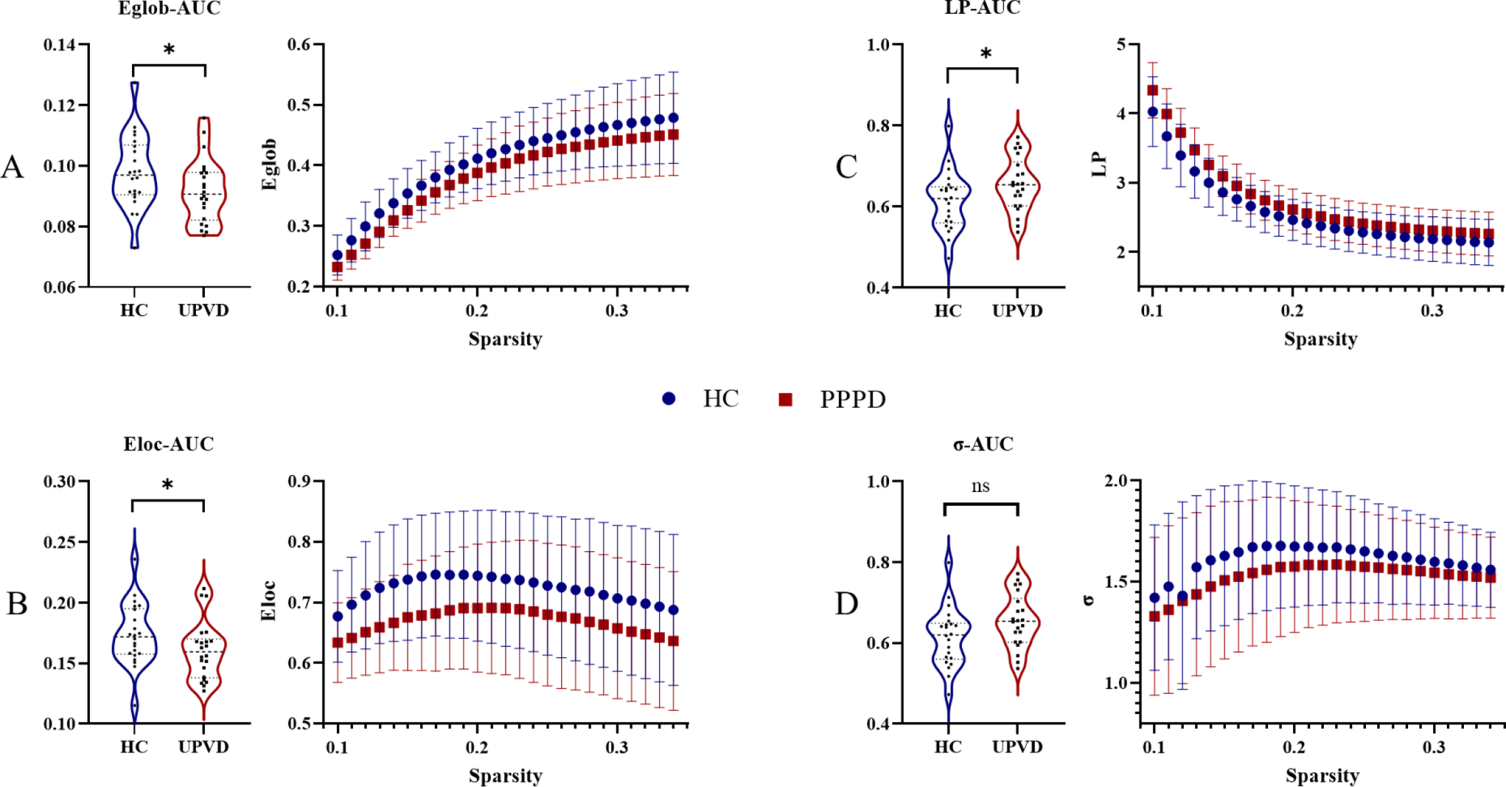
Altered global topological properties of brain network in CUVD patients. **A**: global efficiency (Eglob); **B**: local efficiency (Eloc); **C**: characteristic path length (LP); **D**: small-worldness (σ). CUVP, chronic unilateral vestibulopathy; HC, healthy controls; AUC, area under the curve

Regarding global properties, CUVP patients showed significantly lower global efficiency (T = 3.09, *P* = 0.0052, Fig. 2A) and local efficiency (T = 2.86, *P* = 0.0068, Fig. 2B) compared to healthy subjects. The characteristic path length AUC value was significantly higher in CUVP patients (T=-3.43, *P* = 0.0012, Fig. 2C). Concerning small-world properties (a type of network topology that combines both high clustering (local connectivity) and short path lengths (global connectivity) between nodes), both CUVP patients and healthy subjects had σ values greater than 1, indicating small-world characteristics in both groups. However, no significant differences were observed in small-world properties between the two groups (T = 0.80, *P* = 0.4342, Fig. 2D). The assortativity coefficient (T=-0.99, *P* = 0.3262), CP (T = 1.94, *P* = 0.0582), and modularity coefficient AUC value (T = 0.54, *P* = 0.59) also showed no significant differences between the two groups.

**Fig. 3:**
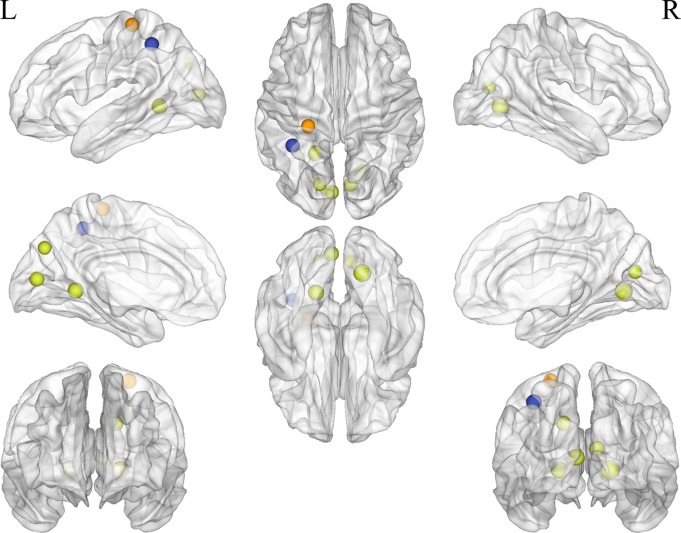
Decreased nodal efficiency in 8 nodes in CUVP patients

Regarding nodal properties, eight nodes showed significantly reduced nodal efficiency (*P* < 0.05, FDR corrected, Fig. 3). Among these, five nodes were located in the visual network: the right cuneus (MNI: X = 9, Y=-76, Z = 14), left BA17 (MNI: X=-5, Y=-80, Z = 9), right lingual gyrus (MNI: X = 19, Y=-66, Z=-1), left occipital BA30 (MNI: X=-18, Y=-50, Z = 1), and left precuneus (MNI: X=-16, Y=-76, Z = 33). Two nodes were located in the fronto-parietal network: the right angular gyrus (MNI: X = 32, Y=-59, Z = 41) and the left intraparietal sulcus (MNI: X=-35, Y=-46, Z = 48). One node was located in the sensorimotor network: the left postcentral gyrus (MNI: X=-24, Y=-30, Z = 64).

**Fig. 4:**
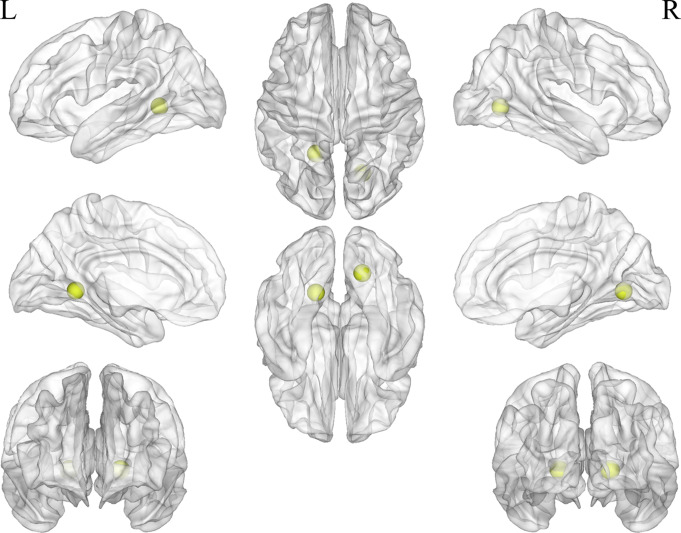
Decreased nodal clustering coefficient efficiency in 2 nodes in CUVP patients

**Fig. 5:**
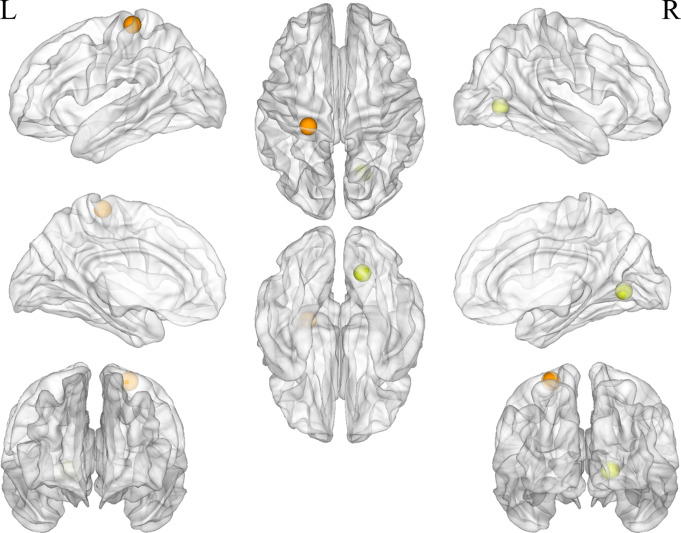
Decreased nodal degree centrality in 2 nodes in CUVP patients

The clustering coefficient of two nodes was reduced (*P* < 0.05, FDR corrected, Fig. 4). These included the left occipital BA30 (MNI: X=-18, Y=-50, Z = 1) and the right lingual gyrus (MNI: X = 19, Y=-66, Z=-1), both located in the visual network. Two nodes showed reduced DC values (*P* < 0.05, FDR corrected, Fig. 5): the right lingual gyrus (MNI: X = 19, Y=-66, Z=-1) and left postcentral gyrus (MNI: X=-24, Y=-30, Z = 64).

### Control for the effect of lesion laterality

#### Network-based statistic analysis

**Fig. 6:**
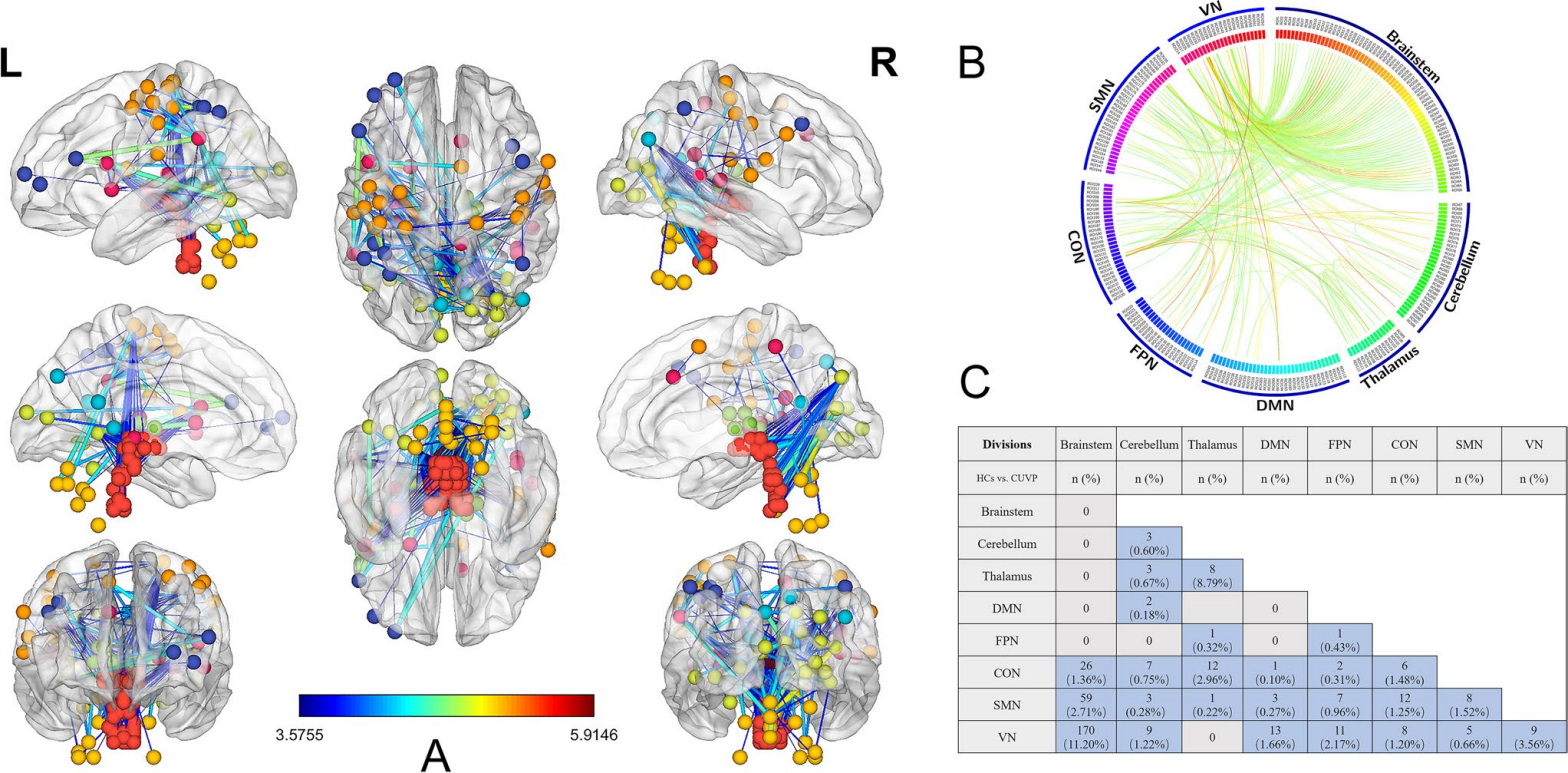
Altered global topological properties of brain network in CUVP patients after controlling for the effect of lesion laterality. **A**: global efficiency (Eglob); **B**: local efficiency (Eloc); **C**: characteristic path length (LP); **D**: small-worldness (σ). CUVP, chronic unilateral vestibulopathy; HC, healthy controls; AUC, area under the curve

After controlling for the effect of lesion laterality, the NBS analysis still revealed an abnormal neural network in CUVP patients (see Fig. 6A), with the parieto-insular vestibular cortex (PIVC), sensory-motor cortex, occipital visual cortex, brainstem, and cerebellum still serving as important nodes in this network. This network included 155 nodes and 390 edges, of which 63 nodes were located in the brainstem, 15 in the cerebellum, 10 in the thalamus, and 67 in the cerebral cortex. The most connections were still found between the brainstem and visual network (VN), with 170 edges. Among the cortical node connections, the cingulo-opercular network (CON) and SMN showed the most significant functional connectivity changes, with 12 edges (1.25% of its inherent connections) (Fig. 6B and C).

#### Graph theoretical analysis

In terms of global properties, the global efficiency (T = 3.1442, *P* = 0.0032) and local efficiency (T = 2.8735, *P* = 0.007) were still significantly lower in CUVP patients compared to healthy subjects. The characteristic path length AUC value in CUVP patients was significantly higher than that of healthy subjects (T=-3.4613, *P* = 0.0014). There was still no significant difference in small-world properties (T = 0.7280, *P* = 0.47), assortativity coefficient (T=-0.9740, *P* = 0.33), CP (T = 1.8720, *P* = 0.067), and modularity coefficient AUC value (T = 0.4651, *P* = 0.6466) between the two groups.

### Correlation analysis results

**Fig. 7:**
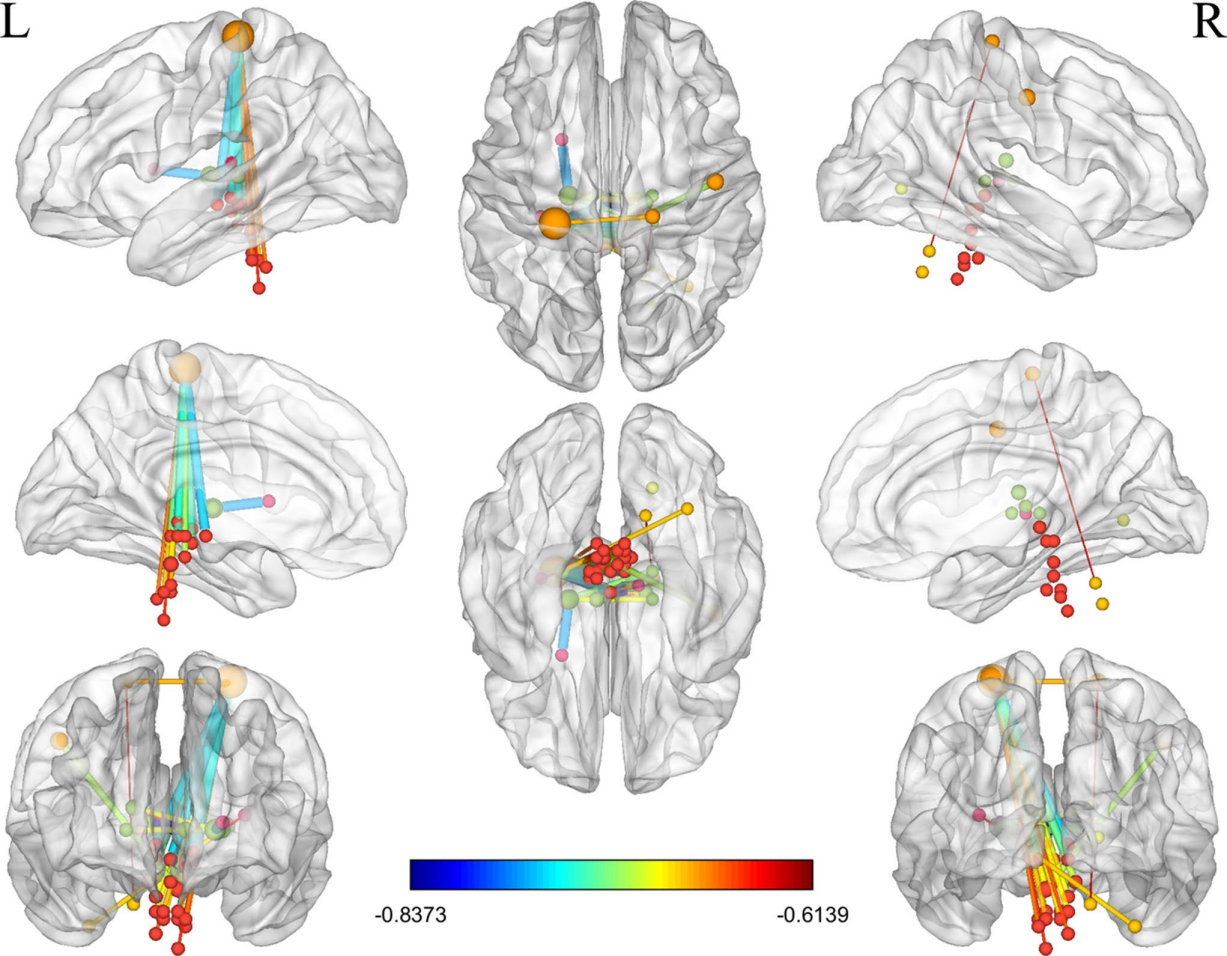
Correlation of the edges in the abnormal neural circuit identified by network-based statistic analysis with the DHI score in CUVP patients

Controlling for age, gender, mean FD as covariates, a total of 47 edges in the abnormal neural circuits identified by NBS analysis showed a negative correlation with DHI (*P* < 0.05, FDR corrected, Fig. 7). Among these, 32 edges were from the brainstem to the sensorimotor network, 5 were internal connections within the thalamus, 3 were thalamus-CON connections, and 3 were between the CON and thalamus. Specifically, 32 edges were the connection between the left postcentral gyrus (MNI: X=-24, Y=-30, Z = 64) and brainstem nuclei.

**Fig. 8:**
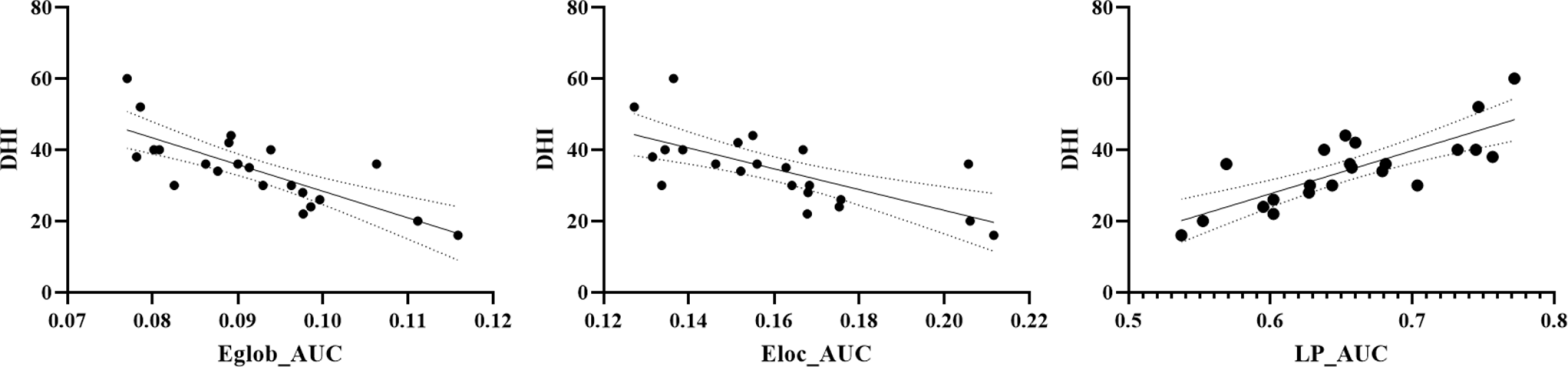
Correlation between the altered topological properties of the brain network and DHI score in CUVP patients. Eglob, global efficiency; Eloc, local efficiency; LP, characteristic path length; AUC, area under the curve; DHI, Dizziness Handicap Inventory score

In terms of brain network topological properties, correlation analysis revealed that global efficiency (*r*=-0.764, *P* < 0.001), local efficiency (*r*=-0.673, *P* = 0.002) in CUVP patients was negatively correlated with DHI scores (Fig. 8). The characteristic path length showed a positive correlation with DHI scores (*r* = 0.771, *P* < 0.001, Fig. 8). There was no significant correlation between CP values and changes in DHI or brain network topological properties.

## Discussion

The vestibular information is a crucial component of the somatosensory system, and damage to vestibular function can lead to both static and dynamic symptoms, with the body compensating through static and dynamic compensation [21]. Although bilateral vestibular nuclei electrical activity can achieve rebalancing during static vestibular compensation, the damage to peripheral vestibular function is often difficult to recover. Vestibular function evaluations, such as the caloric test, still frequently show significant abnormalities [22]. The vestibular information transmitted from the affected peripheral vestibular receptors to the central nervous system remains significantly reduced compared to pre-onset levels or the unaffected side. As a result, vestibular-ocular reflexes and vestibular-spinal reflexes mediated by vestibular information remain abnormal, leading to the persistence of dynamic symptoms. Dynamic compensation occurs more slowly and is often incomplete. CUVP serves as a model for inadequate dynamic compensation. In this study, we further revealed an abnormal neural network in CUVP patients through NBS analysis based on whole-brain functional connectivity. The parieto-insular vestibular cortex, sensorimotor cortex, occipital visual cortex, brainstem, and cerebellum are all key nodes in this network. This finding is crucial for clinicians in understanding brain function abnormalities caused by vestibular dysfunction.

Most previous studies have focused on dynamic compensation at the cortical level [7–9]. Our research indeed identified changes in multiple nodes and interactions between nodes at the cortical level in CUVP patients, with the changes in the cingulo-opercular network, sensorimotor network, and visual network being particularly significant. Our findings indicate that CUVP patients exhibit abnormal interactions among the multi-sensory vestibular cortex, visual network, and sensorimotor network at the cortical level, suggesting that the cortical abnormalities in multi-sensory information may be one of the significant factors contributing to poor vestibular compensation.

Previous studies have lacked sufficient attention to changes in brainstem functional activity in patients with CUVP. It is often assumed that brainstem function returns to normal after static compensation is established, with the focus on the brainstem primarily limited to the bilateral vestibular nuclei [21, 23–24]. Surprisingly, our study found significant changes in interactions between brainstem nuclei and the cortex in CUVP patients. There are three patterns of functional connectivity changes involving the brainstem: brainstem-visual network, brainstem-SMN, and brainstem-CON, with the brainstem-visual network showing the most significant alterations. Previous anatomical studies indicated that vestibular nuclei and other vestibular-related nuclei transmit vestibular information to the cortex via the vestibular nuclei-thalamus-cortex pathway while also receiving feedback from the cortex [25, 26]. Our results show significant functional connectivity abnormalities between the brainstem nuclei and the visual, sensory-motor, and cingulo-opercular networks, suggesting that multi-sensory integration in CUVP occurs not only at the cortical level but also significantly at the brainstem level. Notably, among the 66 brainstem nuclei, 55 exhibited abnormal connections with the cortex, far exceeding our expectations. Vestibular information is a vital part of somatosensory information, and its ascending projections involve interactions among numerous brainstem nuclei, indicating the presence of a vestibular information regulatory network within the brainstem. Previous research on vestibular compensation has primarily focused on the vestibular nuclei. However, with the advancement of studies, other brainstem nuclei, such as the Tuberomammillary nucleus [27], Dorsal raphe nucleus [28], Locus coeruleus [29], and Pedunculopontine tegmental nucleus [30], have been increasingly recognized for their roles in vestibular compensation. Our study reveals that in a model of chronic unilateral peripheral vestibular disease, numerous brainstem nuclei display abnormal functional activity. These findings indicate that after peripheral vestibular damage, the brainstem’s vestibular-related network undergoes reorganization to compensate for or adapt to the reduced vestibular input. This level of complexity in the vestibular compensation network has likely been underestimated in previous studies. More than 50 upstream nuclei have been identified as connected to the medial vestibular nucleus (MVN), primarily located in the hindbrain regions such as the medulla, pons, midbrain, and cerebellum [31–33]. Many of these regions are strongly linked to the MVN and project to its GABAergic and glutamatergic neurons. These findings suggest that vestibular compensation after peripheral vestibular dysfunction is not merely a structural or functional change in a single nucleus or node, but rather a comprehensive reorganization of the entire functional network. Clinically, CUVP presents with different lesion laterality. In this study, there were 13 cases of right-sided vestibular dysfunction and 9 cases of left-sided vestibular dysfunction. In our study, after controlling for the effect of lesion laterality, the results remained significant. This suggests that the changes in brain function and brain networks in CUVP patients reflect impaired compensation due to reduced peripheral vestibular input, rather than differences caused by the side of the lesion.

In this study, we used graph theory analysis to reveal widespread changes in brain network topological properties in CUVP patients. In terms of global properties, CUVP patients exhibited increased characteristic path lengths and decreased local and global efficiency in their brain networks. Global and local efficiency assess how efficiently a network can exchange information, both on a global scale and at the clustering (local) level. Characteristic path length measures the average number of minimum connections needed to connect any two nodes within the network [12, 34]. The human brain, as a complex network with small-world properties, achieves high efficiency with minimal wiring cost. These changes suggest that the efficiency of information transmission is reduced both locally and globally, leading to a state of network disconnection.

In the study by Maximilian Grosch et al. [35], [18 F]-FDG-PET imaging was conducted on rats after unilateral labyrinthectomy (UL). Through graph theoretical analysis, they found increased connectivity, particularly within brain regions linked to the brainstem-cerebellar, thalamocortical vestibular networks, and cortical sensorimotor networks. At the peak of symptoms (day 3 post-UL), brain networks were organized into large clusters of distinct, highly connected hubs, which gradually separated as vestibular compensation progressed. These findings suggest that the brain network changes in patients with CUVP differ from those in patients with AUVP. In the acute phase, relative activation of the contralateral side may play a significant role in brain network alterations, whereas in the chronic phase, the primary feature is a reduction in functional activity due to diminished vestibular input.

Regarding node properties, changes in the node attributes of CUVP patients’ brain networks primarily involved nodal efficiency and clustering coefficients, corresponding with the nodes identified in the abnormal neural networks through NBS. The nodes with altered attributes in CUVP patients were mainly distributed in the visual network, sensorimotor network, and cingulo-opercular network, where nodal efficiency and clustering coefficients decreased, leading to abnormal information transmission between nodes and disrupted integration of visual, vestibular, and proprioceptive information.

For patients with CUVP, the damage to peripheral vestibular function is objectively present, and symptoms such as dizziness appear after the peripheral vestibular injury. Subsequently, due to various factors, the patients remain in a state of chronic decompensation. We hypothesize that the reduction in peripheral vestibular input is the initiating factor for the changes in the patients’ brain networks, reflecting poor compensation for decreased peripheral vestibular input. Abnormalities in multi-sensory integration appear at both brainstem/cerebellar and cortical levels. Correlation analyses confirmed that the changes in brainstem-sensory motor network connectivity and brain network topological properties are associated with clinical symptoms. The lower the efficiency of information transmission in the CUVP patients’ brain networks, the higher their clinical symptom scores. Additionally, it is noteworthy that the changes in brain network topological properties and DHI scores do not significantly correlate with the extent of peripheral vestibular dysfunction. This data is consistent with the study by Palla et al. [36]. This suggests that, after static vestibular compensation restores balance in bilateral vestibular nuclear activity, the severity of clinical symptoms in CUVP patients is more closely related to the efficiency of information transmission following brain network reorganization than to the degree of peripheral vestibular dysfunction.

This study, through NBS analysis and graph theory analysis, reveals the abnormal neural circuits and changes in topological properties of brain networks in CUVP patients. It confirms the presence of multi-sensory integration and information transmission abnormalities at both the brainstem/cerebellar and cortical levels, potentially serving as a functional basis for poor dynamic compensation in patients, which is significant for clinicians to understand the pathophysiological mechanisms related to CUVP. However, this study has certain limitations. First, the sample size is relatively small, and the study is retrospective, a selection bias cannot be excluded. Therefore, prospective studies with larger clinical sample sizes are needed to confirm these findings. In addition, the magnetic fields of MRI scanner may generate Lorentz force that acts on the endolymph, causing endolymph fluid movement. Although all subjects included in the study did not report the occurrence of dizziness or vertigo during MRI scanning, simultaneous eye movement recordings during fMRI would be necessary in future. Furthermore, this study primarily focuses on the differences between CUVP patients with chronic vestibular symptoms and healthy subjects. Although previous studies have shown that the brain function of patients with unilateral peripheral vestibular dysfunction in the recovery phase returns to normal [37, 38], future research is warranted to compare the brain function changes between patients with chronic vestibular symptoms and those who have good central compensation and remain asymptomatic.

## Conclusions

CUVP patients exhibit abnormalities in multi-sensory integration and changes in brain network topological properties at both the brainstem/cerebellar and cortical levels, which may underlie their poor dynamic compensation. It may be interesting to study CUVP patients with different comorbidities in future research to help explore relationship between clinical comorbidities and brain function changes in CUVP patients.

## Electronic supplementary material

Below is the link to the electronic supplementary material.

Supplementary Material 1

## Data Availability

The MRI data that support the findings of this study are available from the corresponding author upon reasonable request.

## Abbreviations

CUVP: Chronic unilateral vestibulopathy
fMRI: Functional magnetic resonance imaging
NBS: Network-Based Statistics
GTA: Graph-theoretical analysis
VBM: Voxel-based morphometry
ALFF: Amplitude of low-frequency fluctuations
ReHo: Regional homogeneity
MNI: Montreal Neurological Institute space
DHI: Dizziness Handicap Inventory score
FDR correction: False discovery rate
PIVC: Parieto-insular vestibular cortex
BA: Brodmanns area
UL: Unilateral labyrinthectomy
MVN: Medial vestibular nucleus
