# Supplementary material for: Abnormal neural circuits and altered brain network topological properties in patients with chronic unilateral vestibulopathy
Source: Neurol Sci. 2025 Apr 21;46(8):3851–63. doi: 10.1007/s10072-025-08183-x (PMC12267322; doi:10.1007/s10072-025-08183-x)
Supplement: Supplementary file 1 — Supplementary Material 1 [file 10072_2025_8183_MOESM1_ESM.docx]

**
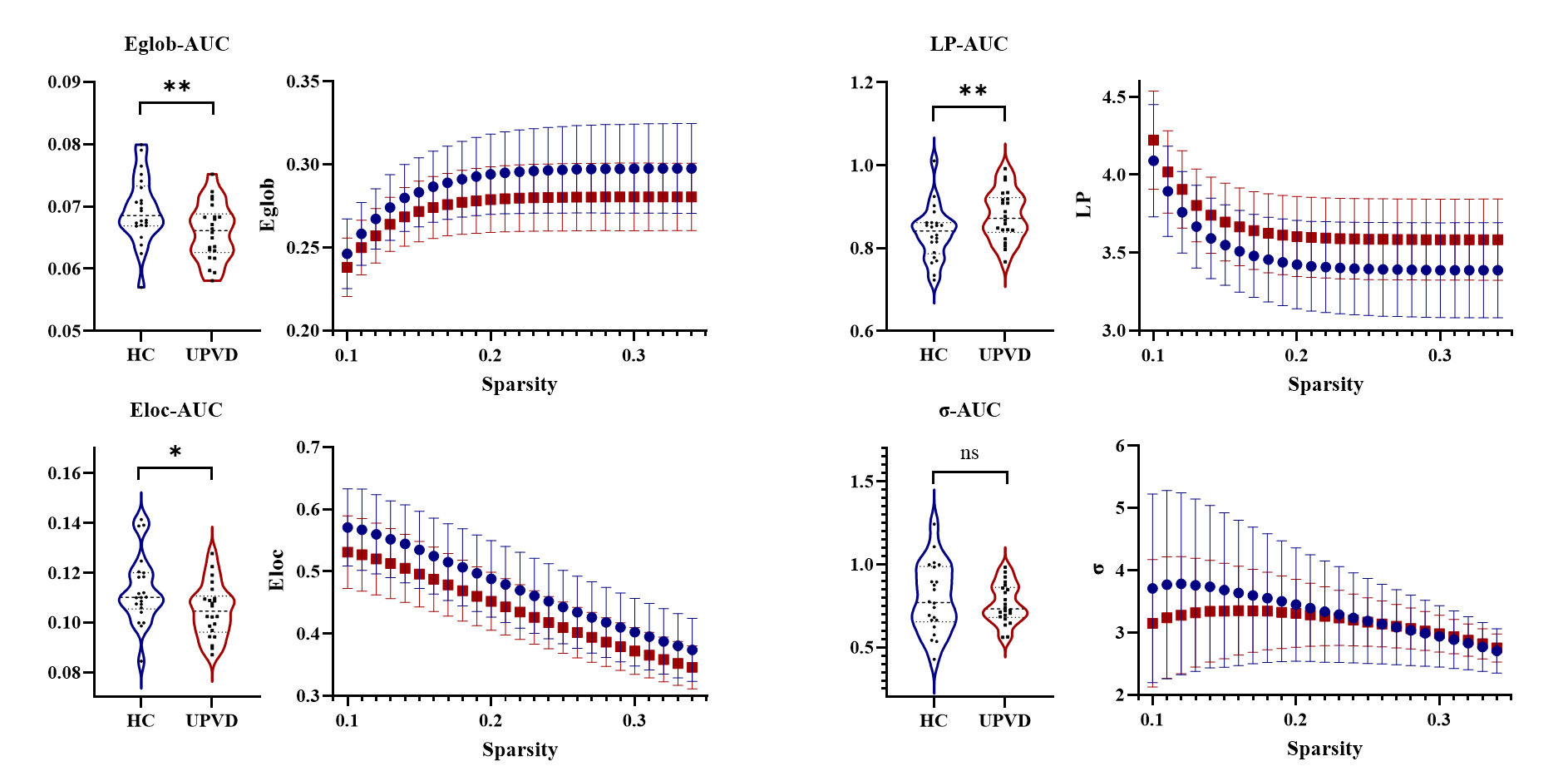
**

**Figure S1** Altered global topological properties of brain network in CUVP patients when regressed out the global mean signal. A: global efficiency (Eglob); B: local efficiency (Eloc); C: characteristic path length (LP); D: small-worldness (σ). CUVP, chronic unilateral vestibulopathy; HC, healthy controls; AUC, area under the curve
